# Supplementation of Nicotinic Acid and Its Derivatives Up-Regulates Cellular NAD^+^ Level Rather than Nicotinamide Derivatives in Cultured Normal Human Epidermal Keratinocytes

**DOI:** 10.3390/life14030413

**Published:** 2024-03-20

**Authors:** Takahiro Oyama, Takumi Yamamoto, Takeshi Kameda, Takanori Kamiya, Hideaki Abe, Takehiko Abe, Sei-ichi Tanuma

**Affiliations:** 1 Hinoki Shinyaku Co., Ltd., 9-6 Nibancho, Chiyoda-ku, Tokyo 102-0084, Japant.kameda@hinoki.co.jp (T.K.); hideaki.abe@aibliss.co.jp (H.A.);; 2 Laboratory of Genomic Medicinal Science, Research Institute for Science and Technology, Tokyo University of Science, 2641 Yamazaki Noda, Chiba 278-8510, Japan; tanuma@rs.tus.ac.jp

**Keywords:** nicotinic acid, NAD^+^, keratinocyte, rejuvenation, inflammaging

## Abstract

Nicotinamide adenine dinucleotide (NAD^+^) plays a pivotal role in various physiological processes within mammalian cells, including energy metabolism, redox homeostasis, and genetic regulation. In the majority of mammalian cellular contexts, NAD^+^ biosynthesis primarily relies on vitamin B3, including nicotinamide (NAM) and nicotinic acid (NA). The concept of NAD^+^ augmentation therapy has recently emerged as a promising strategy to mitigate aging-associated phenomena, termed rejuvenation. Despite the involvement of diverse enzymatic cascades in NAD^+^ biosynthesis, certain cellular environments exhibit deficiencies in specific enzymes, suggesting cell type-dependent variability in optimal NAD^+^ precursor selection. However, the optimization of NAD^+^ precursors for topical formulations has received scant attention thus far. In the present investigation, we sought to delineate the most efficacious precursor for augmenting NAD^+^ levels in human skin keratinocytes. Remarkably, NA supplementation led to a significant 1.3-fold elevation in intracellular NAD^+^ levels, even in the presence of nicotinamide phosphoribosyltransferase inhibition by FK866. Additionally, NA mononucleotide demonstrated a 1.5-fold increase (but not significant) in NAD^+^ levels following 100 μM application. Conversely, NAM and its derivatives failed to elicit a NAD^+^ response in keratinocytes. Notably, NA supplementation elicited up-regulation of mitochondrial superoxide dismutase (SOD2) and sirtuin 3 (SIRT3), indicative of its beneficial impact on mitochondrial function. Furthermore, NA mitigated rotenone-induced mitochondrial reactive oxygen species (ROS) accumulation. Collectively, these findings advocate for the potential utility of NA in topical applications aimed at skin rejuvenation.

## 1. Introduction

Nicotinamide adenine dinucleotide (NAD^+^) is a crucial component for cellular maintenance, including energy metabolism, redox regulation, and gene expression [1,2]. During the aging process, there is a decline in NAD^+^ levels, which has been implicated in the onset and progression of age-related diseases. Low levels of NAD^+^ have been associated with various diseases, such as metabolic and neurodegenerative disorders. Additionally, the decrease in NAD^+^ levels also influences immunomodulation, leading to the polarization of proinflammatory (M1) macrophages, contributing to a phenomenon termed “inflammaging” [1,3]. Considering these findings, NAD^+^ supplementation therapy holds promise for mitigating inflammaging. NAD^+^ supplementation has been shown to increase anti-inflammatory (M2) macrophages, reduce neurodegeneration, activate autophagy and mitophagy, and maintain genome stability, potentially offering health benefits such as decreased inflammation, reduced atrophy, improved brain function, and enhanced insulin sensitivity [1,4,5].

**Figure 1:**
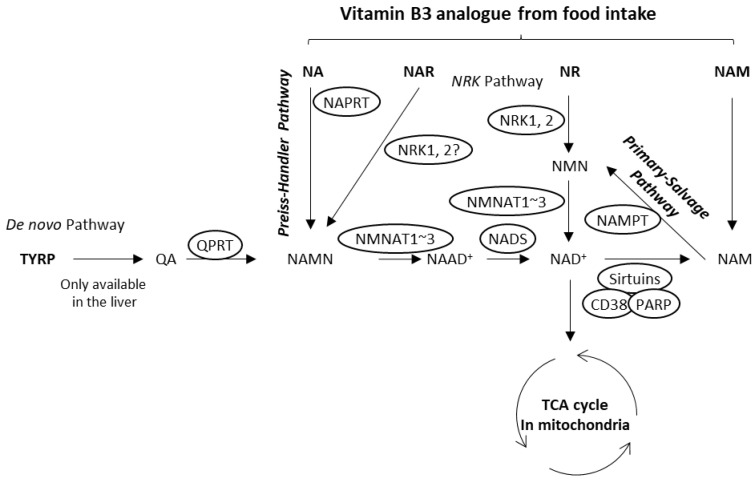
General schemes of the NAD^+^ metabolic pathway. The scheme of the central part of the NAD^+^ metabolic pathway. The enzymes are depicted as circles. Arrows illustrate the synthetic route of NAD^+^. The dotted arrow illustrates the catabolic route of NAD^+^. CD, cluster of differentiation; NA, nicotinic acid; NAD^+^, nicotinamide adenine dinucleotide; NADS, NAD synthetase; NAM, nicotinamide; NAMN, nicotinic acid mononucleotide; NAMPT, nicotinamide phosphoribosyltransferase; NAR, nicotinic acid riboside; NMN, nicotinamide mononucleotide; NMNAT, nicotinamide mononucleotide adenylyl transferase; NR, nicotinamide riboside; NRK, nicotinamide riboside kinase; PARP, poly(ADP-ribose) polymerase; QPRT, quinolinate phosphoribosyltransferase; SIRT, sirtuin; TRYP, tryptophan. ?: unconfirmed part of the pathway.

Primarily, the NAD^+^ synthetic pathway starts with an amino acid, tryptophan (TRYP), called the de novo pathway (Figure 1). TRYP is sequentially metabolized by seven enzymes, although some enzymes are selectively inactivated in human organs [6]. All the enzymes that participate in this pathway are active only in the liver, so peripheral cells like keratinocytes are considered incapable of using the de novo pathway. NAD^+^ produced by the liver is thought to be delivered to peripheral cells by the circulation of the bloodstream. Because NAD^+^ is a large and hydrophilic molecule, it is difficult to penetrate cells. Recently, it has been reported that extracellular NAD^+^ is degraded to nicotinamide (NAM) by the membrane-bound enzyme cluster of differentiation (CD) 73 and that NAM is used as a material for intracellular NAD^+^ production [1,7]. Generally, NAD^+^ production from NAM, called the primary salvage pathway, is the mainstream of NAD^+^ production in peripheral cells. In addition to the primary salvage pathway, the Preiss–Handler pathway is also important for cellular NAD^+^ metabolic pathways. This pathway starts with nicotinic acid (NA). NA is metabolized by nicotinic acid phosphoribosyltransferase 1 (NAPRT1) to nicotinic acid mononucleotide (NAMN) and merged into the de novo pathway. The NAD^+^ produced is used by many proteins that participate in various biological processes, such as sirtuins, CD38, and poly(ADP-ribose) polymerases (PARPs), which result in the liberation of NAM (the pathway is called salvage). Nicotinamide riboside (NR) is an additional salvage precursor that undergoes a two-step [8] or three-step [9] process to form NAD^+^ (Figure 1). This pathway is called the NRK pathway [6,10]. In yeast, NR is metabolized to nicotinic acid riboside (NAR) [9,11]. Recently, the availability of NAR in human cells has been reported [12], but little is known about this molecule. Because NAD^+^ serves as a coenzyme in the tricarboxylic acid (TCA) cycle, maintaining NAD^+^ metabolism is critical for mitochondrial respiration [4,5]. An impairment of oxidative phosphorylation can result in electron leakage, leading to the generation of reactive oxygen species (ROS). Mitochondria, being the primary source of ROS in many cells, possesses a specific ROS removal protein, superoxide dismutase 2 (SOD2). Recent studies have demonstrated that the mitochondria-specific sirtuin, SIRT3, plays a crucial role in deacetylating SOD2, thereby promoting its activity within mitochondria [13,14]. NAD^+^ is directly involved in this system as a substrate for SIRT3 [15], making mitochondria-specific proteins like SIRT3 and SOD2 valuable indicators of mitochondrial health [15,16,17].

Thus far, oral supplementation with nicotinamide mononucleotide (NMN) and NR has been extensively researched and is commercially available for health maintenance [1]. The efficacy of these NAD^+^ precursors varies depending on the cell type [8,18]. However, the most effective precursor for up-regulating cellular NAD^+^ levels in epidermal keratinocytes remains to be fully elucidated.

In this study, we aimed to determine the NAD^+^ precursor suitable for topical application to promote NAD^+^ in epidermal keratinocytes.

## 2. Materials and Methods

### 2.1. Chemical Reagents

NA, NAM, and NAMN were purchased from Sigma (St. Louis, MO, USA). NMN, NR, and NAR were purchased from TCI (Tokyo, Japan), Hangzhou J&H (Hangzhou, China), and Tronto Research Chemicals (Toronto, Canada), respectively. FK866 was purchased from BLD Pharmatech Ltd. (Shanghai, China).

### 2.2. Cells and Cell Culture

Normal human epidermal keratinocytes (NHEK) were purchased from Kurabo (Osaka, Japan: Lot 06657 and 10338) and cultured in a specialized medium (Humedia-KG2) from Kurabo. NHEK is a primary culture of neonatal human keratinocytes. The purchased cells were thawed and pre-cultured at 37 °C in a humidified 5% CO_2_ atmosphere in a standard cell culture flask (Sumitomo Bakelite Co., Ltd., Tokyo, Japan). The cells used for the study were limited to days 5–19 after arriving. The culture medium contains several supplements, such as 0.1 μg/mL human EGF, 0.67 mg/mL hydrocortisone, 10 mg/mL insulin, 50 mg/mL gentamycin, 50 μg/mL amphotericin B, and 2 mL of bovine pituitary gland extract (BPE) in 500 mL Hu-Media KG2, according to the manufacturer’s instructions. According to the information provided by the supplier, Hu-Media KG2 contains 0.3 μM of NAM and does not contain NA.

### 2.3. Measurement of Cellular NAD^+^ Levels

NHEK cells were sub-cultured in a 96-well flat-bottom transparent cell culture plate at 5000 cells/well and pre-cultured for one day. Subsequently, the medium was changed to a new medium containing NAD^+^ precursors at the indicated concentrations. Six hours later, cells were lysed, and the total NAD^+^ amount was measured by the NAD/NADH-Glo™ Assay (Promega Corp., Madison, WI, USA) according to the manufacturer’s protocol. The chemiluminescent signal was measured using SYNERGY HTX (BioTek Instruments, Inc., Winooski, VT, USA).

### 2.4. Immunoblot Analysis

NHEK cells were sub-cultured in a 12-well flat-bottom transparent cell culture plate at 5.0 × 10^4^ cells/well and pre-cultured for one day. After supplementation of NA into the medium, cells were cultured for 24 h. Cells were rinsed with phosphate-buffered saline. The immunoblot analysis was performed as described before [19]. The primary and secondary antibody reactions were performed with an iBind™ Flex Western System (Thermo Fisher Scientific Inc., Waltham, MA, USA). The superoxide dismutase 2 (SOD2) and sirtuin 3 (SIRT3) antibodies were purchased from Cell Signaling Technology, Inc. (Danvers, MA, USA). The glyceraldehyde-3-phosphate dehydrogenase (GAPDH) antibody was purchased from Proteintech, Inc. (Rosemont, IL, USA). The SOD1 protein was detected using an anti-mouse (NA-931V) IgG secondary antibody (GE Healthcare, Chicago, IL, USA). The SOD2 and GAPDH proteins were detected using an anti-rabbit IgG secondary antibody (NA-934V; GE Healthcare).

### 2.5. Mitochondrial Functions

The 1.25 × 10^4^ cells were inoculated into 8-well chamber glass plates. After preincubation, the cells were treated with 1 μM rotenone (Cayman Chemical, Ann Arbor, MI, USA) and/or indicated concentrations of NA. Six hours later, the mitochondrial ROS and mitochondrial membrane potential were measured using mtSOX Deep Red and MitoBright LT Green (Dojindo Laboratories, Kumamoto, Japan), respectively. The fluorescence detection was undertaken by the FLoid™ Cell Imaging Station (Thermo Fisher Scientific Inc.). The quantification of green and red image pixels was conducted by a Python 3 program with Google Collaboratory (Google, Mountain View, CA, USA). The red/green ratio was calculated for each pixel.

### 2.6. Statistical Analyses

All quantitative data are presented as the mean ± standard error (SE). The statistical tests for the difference between two groups and multiple comparisons were conducted using the Student’s *t*-test and one-way ANOVA followed by Dunnett’s test. The significance level was set as α = 0.05.

### 2.7. Declaration of Generative AI in Scientific Writing

During the preparation of this work, the authors used ChatGPT: model architecture GPT-3.5 (Open AI, San Francisco, CA, USA) in order to paraphrase and brush up expressions. In addition, we used Trinka (Crimson AI Pvt. Ltd., Mumbai, India) for language editing. After using this tool/service, the authors reviewed and edited the content as needed and take full responsibility for the content of the publication.

## 3. Results

### 3.1. Nicotinic Acid Activates NAD^+^ Production in Normal Human Epidermal Keratinocytes

**Figure 2:**
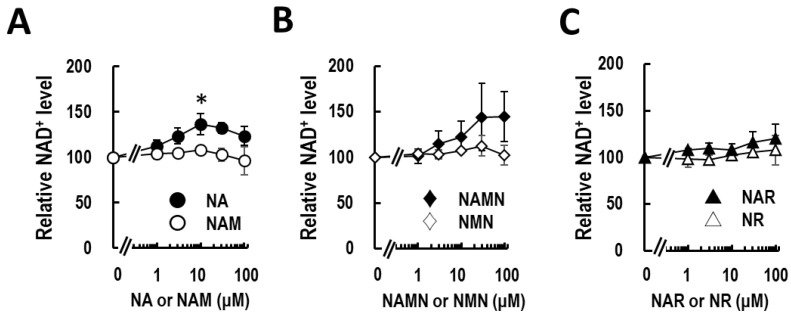
Nicotinic acid activates NAD^+^ production in cultured keratinocytes. The dose-dependent effects of NA and NAM (**A**), NAMN and NMN (**B**), and NAR and NR (**C**) supplementation on NAD^+^ in NHEK cells. The concentrations of the compounds were 1, 3, 10, 30, and 100 μM. The statistical analyses were performed by a one-way ANOVA followed by Dunnett’s test. *: *p* < 0.05 vs. 0. All data are expressed as the mean ± SE of at least three independent experiments. NA, nicotinic acid; NAD^+^, nicotinamide adenine dinucleotide; NAM, nicotinamide; NAMN, nicotinic acid mononucleotide; NAR, nicotinic acid riboside; NMN, nicotinamide mononucleotide; NR, nicotinamide riboside.

Mammalian bodies utilize three metabolic pathways for the production of NAD^+^. However, the availability of these pathways is different for different cell types (Figure 1) [8,18]. To date, knowledge about which pathway is used by cultured human keratinocytes is limited. To address this question, we evaluated six known NAD^+^ precursors (NA, NAMN, NAR, NAM, NMN, and NR) for the NAD^+^ production assay. The incubation time was determined to be 6 h by preliminary studies (Supplementary Figure S1) and a report by Hara et al. [20], which shows that at least 2 h after supplementation with NA can up-regulate NAD^+^ levels in HEK293 cells. As shown in Figure 2A, NA significantly up-regulated the cellular NAD^+^ level by 1.3-fold at 10 μM, but high-dose NA supplementation (30–100 μM) slightly reversed the effect. NAMN also up-regulated the NAD^+^ level substantially by 1.5-fold at 30 and 100 μM (but not statistically significantly; Figure 2B). NAR could regulate the NAD^+^ level slightly positively and showed dose dependency, with a 1.1-fold increase at 100 μM (Figure 2C). In contrast, NAM, NMN, and NR could not increase the NAD^+^ level (Figure 2). These results indicate that epidermal keratinocytes can use the Preiss–Handler pathway under NA- or NAMN-sufficient conditions.

### 3.2. Nicotinic Acid-Recovered FK866-Induced NAD^+^ Depletion in Normal Human Epidermal Keratinocytes

**Figure 3:**
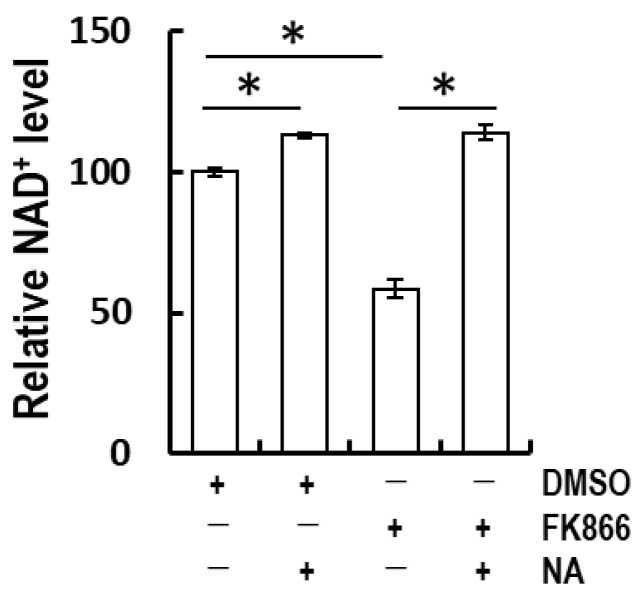
Nicotinic acid-recovered FK866-induced NAD^+^ depletion in cultured keratinocytes. Recovery of the depletion of NAD^+^ levels by pre-treatment with 1 nM FK866 for 24 h in NA-supplemented medium. After FK866 treatment, 10 μM NA was treated for 6 h. The statistical analyses were performed by the Student’s *t*-test. * *p* < 0.05. The data are expressed as the mean ± SE of at least three independent experiments.

To validate the functionality of the Preiss–Handler pathway, cellular NAD^+^ levels were assessed under conditions of NAMPT blockade induced by 1 nM FK866 [21] treatment with or without NA supplementation. FK866 treatment halved cellular NAD^+^ levels, which were subsequently restored to unblocked levels by NA treatment (Figure 3). These findings suggest that keratinocytes primarily utilize the primary salvage pathway, but the Preiss–Handler pathway can serve as a compensatory route for NAD^+^ production.

### 3.3. Nicotinic Acid Supplementation Ablates Rotenone-Induced Mitochondrial ROS in Normal Human Epidermal Keratinocytes

**Figure 4:**
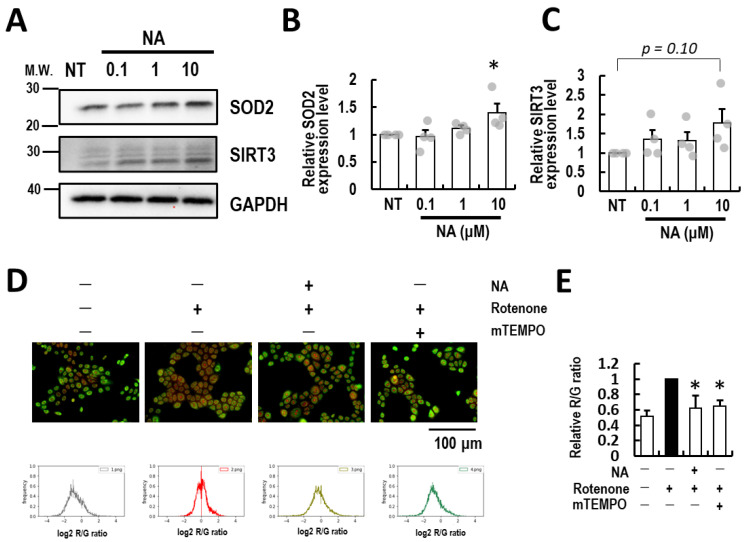
Ablation of mitochondrial ROS by the supplementation of nicotinic acid (**A**) Representative images of the effects of NA on the mitochondrial markers superoxide dismutase (SOD) 2 and sirtuin 3 (SIRT3). Glyceraldehyde 3-phosphate dehydrogenase (GAPDH) is shown as the loading control. (**B**,**C**) Quantification of SOD2 (**B**) and SIRT3 (**C**) immunoblot bands on (**A**). The data are divided by the intensity of GAPDH. Individual quantified values are represented as gray dots on the bar chart. (**D**) Representative images of the effect of NA on mitochondrial ROS recovery activity. Mitochondria and mitochondrial ROS were stained by green and red fluorescent dye, and 50 μM mito-TEMPO was used as a positive control. R: 1 μM rotenone. The red/green pixel ratio (R/G ratio) is depicted by a histogram, and the means of the histograms are shown in (**E**). All data are expressed as the mean ± SE of at least three independent experiments. The statistical analyses were performed by a one-way ANOVA followed by Dunnett’s test. * *p* < 0.05.

Because NAD^+^ is a carrier of electrons to mitochondria, NAD^+^ up-regulation is thought to affect mitochondria. We monitored mitochondrial SOD2 and SIRT3 as markers of mitochondrial status. As a result, the SOD2 and SIRT3 protein levels were up-regulated by NA supplementation (Figure 4A–C: *p* = 0.04 and 0.10, respectively). Next, we assessed the effect of NA on mitochondrial dysfunction. The mitochondrial complex I inhibitor rotenone [13] was used as the inducer of mitochondrial ROS. As a result, NA supplementation also reduced rotenone-induced mitochondrial ROS production (Figure 4D,E) as well as the mitochondria-targeted radical scavenger mito-TEMPO [22]. These results indicate that NA supplementation can be resistant to mitochondrial damage in keratinocytes.

## 4. Discussion

Vitamin B3, including NA and NAM, is a pivotal nutrition as precursors of NAD^+^. Deficiency in this vitamin leads to a significant skin disorder known as pellagra [1]. NAD^+^ was first discovered in 1906 by Arthur Harden as a required small molecule for alcoholic fermentation, and now we know that NAD^+^ is one of the key regulators of various cellular processes [23]. Conrad Elvehjem discovered vitamin B3 (NA and NAM) as a remedy for pellagra, which is a severe skin disease, in an era where nutritional science was lacking [24]. Simultaneously, Otto Warburg and Hans von Euler connected vitamin B3 to its bioactive form, NAD^+^. Then, NAD^+^ metabolism was recognized as an important field in health research. In the second half of the century, another vitamin B3, NR, was discovered by Charles Brenner [8,25]. Recently, NAD^+^ supplementation therapy has improved various impairments in our bodies caused by aging. In particular, NMN and NR attract our attention to achieve rejuvenation because many preclinical experiments and clinical trials have been conducted, and some of the studies successfully improved body function in both mouse and human aging models [1,4,5,10,26].

Outside the liver, most cells do not express the full array of enzymes necessary to convert TRYP to NAD^+^ by the de novo pathway [1]. Many types of cells properly select the pathways in each environment to guarantee the pivotal currency of energy, i.e., neurons use a part of the de novo and the NRK pathways, adipocytes only use the salvage pathway, immune cells use a part of the de novo and the salvage pathway, and intestinal epithelium uses the Preiss–Handler and the salvage pathways [3]. In line with this context, an appropriate selection of NAD^+^ precursors for up-regulating cellular NAD^+^ levels in keratinocytes should be considered. Gensler et al. revealed that oral administration of NA at levels that increase skin NAD^+^ content inhibits UV-induced carcinogenesis and photoimmune suppression in an animal model [27]. However, the ingredients that are necessary and sufficient for topical applications, such as lotion, cream, and ointment, are poorly understood. In this study, the NAD^+^ level was markedly up-regulated by the supplementation of NA and slightly by NAMN, which indicates that the Preiss–Handler pathway is active in NHEK cells.

According to the supplier, the NAM content in the cultured medium is 0.3 μM. To sum up our data and this information, 0.3 μM NAM is sufficient for the maintenance of cellular physiological conditions, partly because of its effective recycling system of NAD^+^. However, the Preiss–Handler pathway is active but not used in normal conditions of culture because no NA derivatives are supplemented in the culture medium. Compared to the upper limit of NAM (0.3 μM), the upper limit of NA is about 10–30 μM (Figure 2A). These results suggest that NA is suitable as topical supplementation for NAD^+^ up-regulation therapy for human keratinocytes compared to NAM and NMN. Figure 2A also shows a slight decrease in NAD^+^ levels in 6 h after treatment with NAM. NAM is known as a negative regulator of SIRT1, which is the key component of the circadian clock [28,29]. The negative feedback loop may be related to our observation that NAM supplementation causes a slight decrease in cellular NAD^+^ levels. It is worth noting that there might be unexplored effects of these precursors on cells at different time points. Future research should investigate NAD^+^ availability at different time points, not only in the short term (6 h). However, the influence of circadian variation should be considered, as cellular NAD^+^ levels exhibit rhythmic changes throughout the day [30].

In general, ionic molecules are hard to permeate into the cell membrane. To transport these molecules into cells, cells express ionic transporters, like solute career proteins (SLCs). To date, studies have discovered the transporters of NA and NMN to be SLC5A8/SLC22A13 and SLC12A8, respectively [1]. Recently, NR and NAR have been demonstrated to be imported into HEK293 cells via the SLC28 and SLC29 families [12]. The availability of NAD^+^ precursors may depend on not only the expressions of NAD^+^-producing enzymes but also these transporter proteins in certain cells. In this study, NAR and NR had little effect on the cellular NAD^+^ level compared with NA and NAMN (Figure 2). According to the analysis of Fujisawa et al., SLC28 was expressed in the skin at a low level compared to that in the liver or small intestine [31]. This observation is consistent with our research, but a more detailed study is necessary.

To date, little is known about the carrier protein responsible for introducing NAMN into cells. In our data, NAMN showed a tendency to up-regulate cellular NAD^+^ levels, suggesting the existence of an unknown route for transporting NAMN into cells. Although the effect was observed, there was considerable variance, possibly attributable in part to the molecule’s stability. However, there have been no studies on the stability of extracellular NAMN under physiological conditions. For topical applications, NAMN may also emerge as a candidate for a new ingredient in cosmetics, but further research is needed, as mentioned above.

Most in vivo studies on NMN treatment involve oral gavage and intraperitoneal injection. Zhou et al. demonstrated that both forms of administration of NMN protect against UVB-induced skin damage in mice [32]. However, NMN supplementation in the culture medium did not directly up-regulate NAD^+^ levels in our study. Thus, NMN may indirectly protect against skin damage. In the future, in vivo studies on topical application to UVB-damaged skin should be conducted to confirm the results of the present study.

In the present study, the supplementation of NA up-regulated SOD2 (*p* < 0.05) and SIRT3 (*p* = 0.10) proteins, which indicates that NAD^+^ reservation therapy affects the mitochondrial anti-oxidative potential (Figure 4A,C). During aging processes, NAD^+^-consuming enzymes increase, which leads to mitochondrial ablation and cellular senescence [1,4,5]. Additionally, the deterioration of mitochondrial metabolism in T cells imbalances macrophage types toward a pro-inflammatory M1 state. This process is called inflammaging, and it should be evaded [1]. The up-regulation of SOD2 protein level might have removed mitochondrial superoxide (Figure 4D,E), resulting in inhibition of these silent changes in the mitochondria in keratinocytes. 

Generally, NAM is used for topical applications for removing wrinkles and moisturizing [33]. Conversely, in this study, NA was found to increase NAD^+^ levels in epidermal keratinocytes, potentially enhancing skin barrier function through anti-aging mechanisms. Therefore, effective methods for maintaining healthy skin conditions should be combined according to specific goals.

Note that NA administration carries a risk of niacin flushing, characterized by cutaneous vasodilation accompanied by tingling and burning sensations [34,35]. Recently, the NA receptor GPR109A was discovered in adipocytes and skin Langerhans cells as the cause of niacin flushing [36]. Hanson et al. revealed that keratinocytes also express GPR109A and are involved in NA and monomethyl fumarate-induced flushing and COX2-dependent prostanoid formation in mice [37]. In our results in Figure 2A, supplementation with at least 30 μM NA slightly decreased the NAD^+^ level compared to a 10 μM application. This phenomenon might be related to GPR109A-induced flushing. Despite this concern, NA has been utilized in cosmetic formulations in the range of 0.01% to 0.1% (approximately 8–80 mM eq) in several products in the USA [38]. Toxicity report analyses by the Cosmetic Ingredient Review Expert Panel have revealed that both niacinamide and niacin are safe at current usage and concentration levels in cosmetic products [38]. In the future, it is essential to estimate whether the effect of NA within a safe dosage range can impact cellular NAD^+^ levels in vivo.

## 5. Conclusions

This study demonstrates that keratinocytes can utilize the Preiss–Handler pathway. Supplementation with NA increases cellular NAD^+^ levels and can reduce oxidative stress in mitochondria. In conclusion, NA is a good material for maintaining the physiological conditions of skin keratinocytes.

## Data Availability

The raw data supporting the conclusions of this article will be made available by the authors on request.

## Supplementary Materials

The following supporting information can be downloaded at https://www.mdpi.com/article/10.3390/life14030413/s1, Figure S1: Preliminary study of NAD^+^ precursor supplementation in keratinocytes. Figure S2: Original bolts.
