# Supplementary material for: Supplementation of Nicotinic Acid and Its Derivatives Up-Regulates Cellular NAD+ Level Rather than Nicotinamide Derivatives in Cultured Normal Human Epidermal Keratinocytes
Source: Life (Basel). 2024 Mar 20;14(3):413. doi: 10.3390/life14030413 (PMC10971338; doi:10.3390/life14030413)
Supplement: Supplementary file 1 [file life-14-00413-s001.zip › Supplemental Figure S1.pdf]

Figure S1

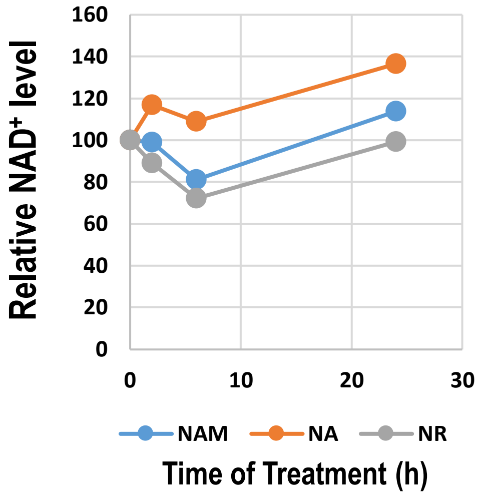

Supplemental Figure 1: Preliminary study of NAD+ precursor supplementation in keratinocytes.

To investigate the temporal changes in cellular NAD+ levels upon supplementation with NAM, NA, and NR, 10 μM of these precursors were added to the culture medium of NHEK cells, and NAD+ levels were measured at 2, 6, and 24 hours post-supplementation. As depicted in Supplementary Figure 1, NA supplementation markedly increased NAD+ levels by 1.2-fold at 2 hours compared to baseline, and this elevation was sustained up to 24 hours. In contrast, NAM and NR application initially decreased NAD+ levels to 0.8-fold at 6 hours, followed by a recovery to baseline levels by 24 hours. Initially, we found this contrast intriguing, wherein NA could upregulate NAD+ while NAM and NR transiently suppressed NAD+ production. Consequently, we decided to compare NAD+ levels at 6 hours not only for NA, NAM, and NR but also for other precursors (NMN, NAMN, and NAR). However, as illustrated in Figure 2, NAM and NR supplementation did not significantly decrease NAD+ levels, possibly due to the averaging of data from three independent experiments mitigating errors or circadian fluctuations. Therefore, we further investigated the effect of NA in subsequent experiments.
