# Supplementary figures and images for: Supplementation of Nicotinic Acid and Its Derivatives Up-Regulates Cellular NAD+ Level Rather than Nicotinamide Derivatives in Cultured Normal Human Epidermal Keratinocytes
Source: Life (Basel). 2024 Mar 20;14(3):413. doi: 10.3390/life14030413 (PMC10971338; doi:10.3390/life14030413)

# Original Blots

## SOD2

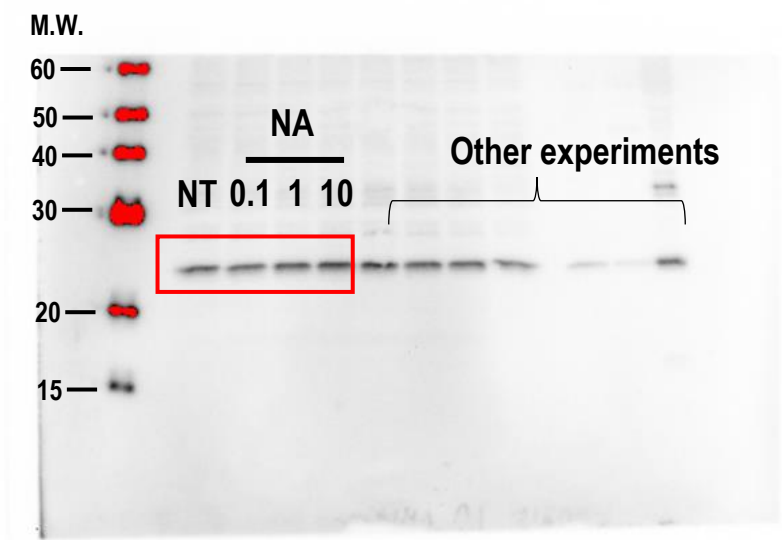

## SIRT3

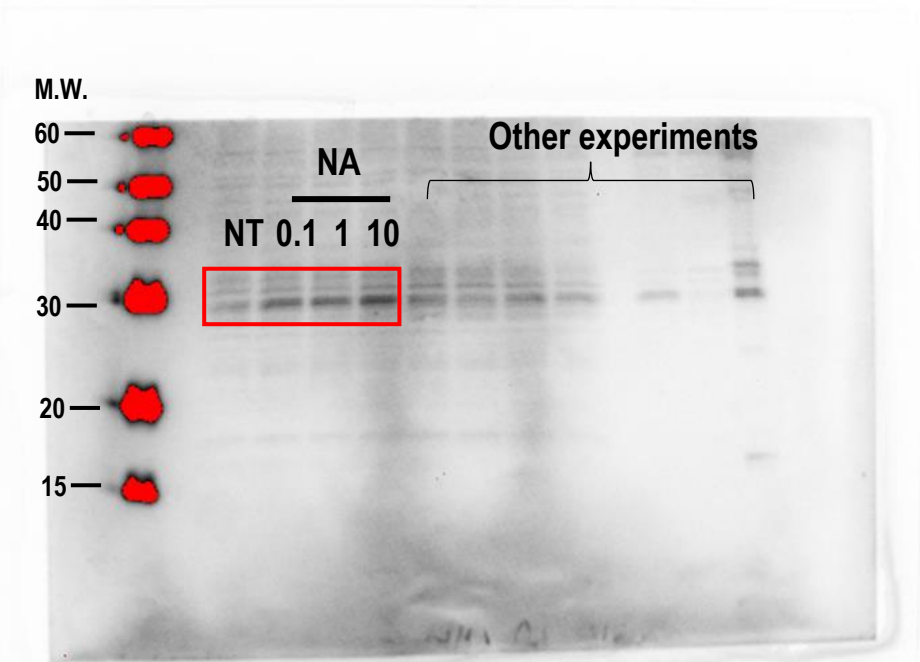

GAPDH

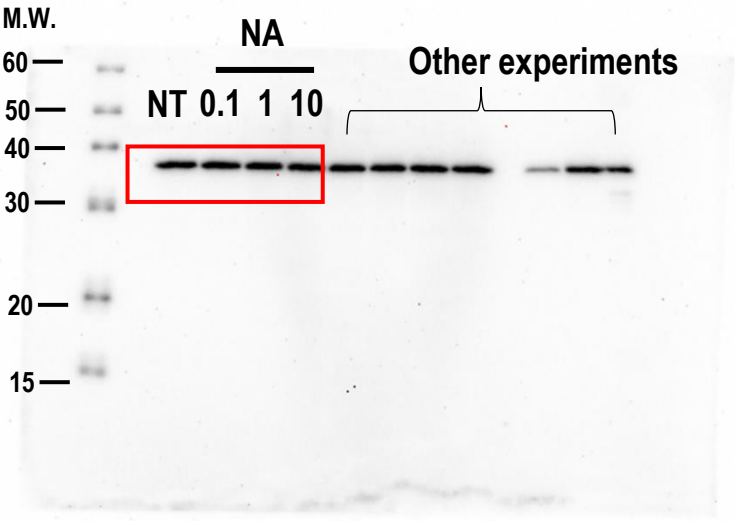

Supplement: Supplementary file 1 [file life-14-00413-s001.zip › Original Blots (Supplemental Figure S2).pdf]
